# Associations between Length of Stay in Long Term Care Facilities and End of Life Care. Analysis of the PACE Cross-Sectional Study

**DOI:** 10.3390/ijerph17082742

**Published:** 2020-04-16

**Authors:** Danni Collingridge Moore, Sheila Payne, Thomas Keegan, Luc Deliens, Tinne Smets, Giovanni Gambassi, Marika Kylänen, Violetta Kijowska, Bregje Onwuteaka-Philipsen, Lieve Van den Block

**Affiliations:** 1 International Observatory on End of Life Care, Lancaster University, Lancaster LA1 4YW, UK; s.a.payne@lancaster.ac.uk; 2 Lancaster Medical School, Lancaster University, Lancaster LA1 4YG, UK; t.keegan@lancaster.ac.uk; 3 VUB-UGhent End of Life Care Research Group, Vrije Universiteit Brussel (VUB), 1090 Brussels, Belgium; Luc.Deliens@vub.be (L.D.); Tinne.Smets@vub.be (T.S.); lieve.van.den.block@vub.be (L.V.d.B.); 4 Department of Geriatrics and Orthopaedic Sciences, Università Cattolica del Sacro Cuore, 00168 Rome, Italy; Giovanni.Gambassi@unicatt.it; 5 National Institute for Health and Welfare, (00)271 Helsinki, Finland; marika.kylanen@thl.fi; 6 Unit for Research on Aging Society, Department of Sociology, Faculty of Medicine, Jagiellonian University Medical College, 31-034 Krakow, Poland; viola.kijowska@gmail.com; 7 Amsterdam UMC, Vrije Universiteit Amsterdam, Department of Public and Occupational Health, Amsterdam Public Health research institute, Expertise Center for Palliative Care, 1081 HV Amsterdam, The Netherlands; b.philipsen@vumc.nl

**Keywords:** long-term care facility, care home, nursing home, length of stay, palliative care, end of life care, epidemiology

## Abstract

Long term care facilities (LTCFs) are increasingly a place of care at end of life in Europe. Longer residence in an LTCF prior to death has been associated with higher indicators of end of life care; however, the relationship has not been fully explored. The purpose of this analysis is to explore associations between length of stay and end of life care. The analysis used data collected in the Palliative Care for Older People in care and nursing homes in Europe (PACE) study, a cross-sectional mortality follow-back survey of LTCF residents who died within a retrospective 3-month period, conducted in Belgium, England, Finland, Italy, the Netherlands and Poland. Primary outcomes were quality of care in the last month of life, comfort in the last week of life, contact with health services in the last month of life, presence of advance directives and consensus in care. Longer lengths of stay were associated with higher scores of quality of care in the last month of life and comfort in the last week of life. Longer stay residents were more likely to have advance directives in place and have a lasting power of attorney for personal welfare. Further research is needed to explore the underlying reasons for this trend, and how good quality end of life care can be provided to all LTCF residents.

## 1. Introduction

Long-term care facilities (LTCFs) are becoming a common place of death for older adults [1,2,3], especially those with dementia [4,5]. Although terminology and typology varies between countries, a LTCF, including care homes and nursing homes, generally refers to a collective institutional setting where care is provided to older adults, who live there, 24 h a day, seven days a week [6].

Ensuring LTCF residents approaching end of life receive appropriate care is challenging; residents may be frail, with multiple, complex care needs, and may be unable to either establish or communicate their preferences at end of life. Long term care facilities are often staffed by a combination of registered, qualified nurses and care assistants, who may have limited knowledge of end of life care for older adults and limited access to specialist services to support end of life care [7]. In addition, LTCF managers and their staff may lack clarity in defining and identifying end of life, or their role or responsibility in providing subsequent care within the facility [8]. In many European countries, end of life care in LTCFs in not well supported at a national level; in a review of 29 countries only eight had national policies which specifically addressed end of life care in LTCFs [9]. 

Palliative care is defined as “an approach that improves the quality of life of patients and their families facing the problem associated with life-threatening illness, through the prevention and relief of suffering by means of early identification and impeccable assessment and treatment of pain and other problems, physical, psychosocial and spiritual” [10]. The term “end of life care” is often used synonymously with palliative care in the UK, and refers to “an extended period of 1 to 2 years during which the patient/family and health professionals become aware of the life-limiting nature of their illness” [11]. Previous studies have found that the adoption of a palliative care approach in LTCFs led to a reduction in deaths outside the LTCF [12], an increase in the numbers of completed advance directives [13], improvements in end-of-life communication between residents, relatives and health professionals [14,15,16] and improvements in staff knowledge [17,18,19].

Numerous interventions have been developed to improve the provision and quality of end of life care in LTCFs, including staff education [20,21], inter-professional collaborations and care coordination [22,23], either through individual initiatives or as part of multicomponent interventions, such as the Liverpool Care Pathway [24], Gold Standards Framework for Care Homes [25] or the Steps to Success intervention [26]. The time point at which these initiatives aim to change the care provided to a resident varies; whereas the Liverpool Care Pathways focuses on care in the last days of life [24], interventions focusing on communicating preferences at end of life may be introduced either at admission or four to six weeks post admission. For residents who die shortly after admission, such activities may occur simultaneously.

Although specific guidelines exist for providing end of life care specifically to older adults [27] and those with dementia [28], less research has explored variation in the palliative care delivered to specific subgroups, such as women or older adults with little support from family carers. In particular, it is unclear whether the end of life care received by residents admitted shortly before death differs from the care for those who have lived in a facility for many months or even years [29]. Previous studies exploring care at end of life have found that residents with longer length of stay before death had fewer hospitalisations, were more likely to be receive palliative drug therapy, less likely to be undertreated for non-pain symptoms and more likely to have documented do-not-resuscitate (DNR) orders in place [30,31,32,33]. 

At present, no published research has specifically explored the association between length of stay in a LTCF and the experience of residents at end of life, collected either directly from the resident or by proxy measures. None of the research previously discussed included length of stay as a primary explanatory variable of the end of life care indicators investigated, and none report conducting any prior analysis to explore factors associated with length of stay in the data. Therefore, previous research findings may not control for all characteristics associated with longer lengths of stay, leading to associations between end of life care and resident characteristics, such as age, gender, dementia diagnosis or marital status, being confused with associations with length of stay. In addition, it is common for LTCF residents to fall into one of two broad populations, those with relatively short stays before death and those who have resided in the facility for many years [8]. In previous analysis of length of stay and end of life care in LTCFs, residents with different lengths of stay have commonly been separated into residents residing in the facility either 6 months, 1 year or 2 years before death, leaving the experience of residents with longer lengths of stay unexplored. 

A greater understanding of how the experience of residents at end of life varies is a research priority, and can inform the development of interventions aiming to improve the provision and quality of end of life care in LTCFs, and explore variation within a heterogeneous population. In this analysis we used data from the Palliative Care for Older People in care and nursing homes in Europe (PACE) cross-sectional study, which aimed to compare quality of dying and end of life care in deceased residents of LTCFs in six European countries [34]. The purpose of this analysis is to explore whether length of stay in LTCF residents is related to five indicators of end of life care; quality of care in the last month of life, comfort in the last week of life, contact with health services in the last month of life, presence of advance directives and consensus among those involved in care and treatment, using staff-reported data on deceased residents from LTCFs in six European countries. 

## 2. Materials and Methods 

### 2.1. Study Design and Setting 

The data used in this analysis are from a cross-sectional, mortality follow back survey of deceased residents; the PACE study [35]. The PACE study was conducted in a sample created, where possible, using national lists of LTCFs in Belgium, England, Finland, Italy, the Netherlands and Poland, recruited using a proportionally stratified random sampling framework [36].

In LTCFs that consented to take part in the study, data were collected on residents who had died in a 3-month retrospective period during 2015. Residents were included in the study if they had died in the facility or after transfer to hospital. For each identified resident, demographic information was collected from either administrative staff or the facility manager (response rate 95.7%), and a postal questionnaire sent to a LTCF staff member regarded as most involved in the resident’s care (81.6%). A full description of the study methodology, including ethical approvals, are described elsewhere [35]. 

### 2.2. Measurements

A LTCF staff member (nurse or care assistant), identified by a key person appointed by the LTCF manager as most involved in the residents’ care, self-reported the main outcomes used in this analysis. Data were collected on (i) quality of care in the last month of life, (ii) comfort in the last week of life, (iii) contact with health services in the last month, (iv) presence of advance directives, and (v) consensus among those involved in care and treatment. 

Quality of care in the last month of life (i) was measured using the Quality of Dying in Long-Term Care (QoD-LTC) scale [37]. The questionnaire has 11 items, with higher scores indicating better quality of care. Three subscales, personhood, closure and preparatory tasks, can be generated. Comfort in the last week of life (ii) was measured using the End-of-Life in Dementia Scale Comfort Assessment While Dying (EOLD-CAD) scale [38]. The questionnaire has 14 items, with higher scores indicating higher levels of comfort. Four subscales, physical distress, dying symptoms, emotional distress and wellbeing, can be calculated. 

The data on contact with health services at end of life (iii) were number of visits either received or made by a physician during the last month of life, number of admissions to a hospital, geriatric ward, intensive care unit or general ward (for more than 24 h) during the last month of life, and the number of visits to a hospital emergency room (for less than 24 h) during the last month of life. Resident’s place of death was categorised as either death in a LTCF or in a hospital.

The presence of advance directives (iv) was determined using four outcomes. Firstly, whether the resident had any written advance directives in place, including a do not resuscitate in case of a cardiac or respiratory arrest order, do not transfer to a hospital order, a request to discontinue the use of, or do not use, other treatments, or a request to try all life sustaining measures. Secondly, whether the resident had a lasting power of attorney for personal welfare. Thirdly, whether a staff member ever spoke with the resident about medical treatments he or she would or would not want in the last phase of life or about the preferred course of care in the last phase of life. The final outcome was whether a staff member spoke with a relative of the resident about medical treatments he or she would or would not want in the last phase of life or about the preferred course of care in the last phase of life, prior to a decision being made.

The degree to which those involved in care were in agreement (consensus) on care and treatment in the last month of the resident’s life (v), from the perspective of staff members, was measured among LTCF staff, among representatives/family and among all those involved in the resident’s care. Staff members were asked to select one of three choices for each question; full consensus, consensus on major issues or no consensus. In this analysis, the answers were categorised as consensus (full consensus or consensus on major issues) or no consensus (no consensus).

Length of stay was calculated in days using date of admission to the LTCF and date of death. Residents were grouped based on their lengths of stay in seven groups: under 1 month, 1 to 3 months, 3 months to 1 year, 1 to 2 years, 2 to 3 years, 3 to 5 years and over 5 years. The groups were demarcated to ensure relatively similar sample sizes in each group, and to allow analysis of longer stay residents. Ten variables previously identified as associated with length of stay in the dataset were included in the analysis to control for resident, LTCF and country characteristics [39]. These were age, gender, marital status, place of admission, presence of cancer, presence and severity of dementia, physical functioning, LTCF type, LTCF funding status and country. 

Age and gender were determined at the time of admission. Severity of dementia was calculated using a combined score from the Global Dementia Scale (GDS) [40] and the Cognitive Performance Scale (CPS) [41]. The Bedford Alzheimer Nursing-Severity Scale (BANS-S) [42] was used to measure physical functioning.

Each LTCF was categorised by the type of care offered, as type 1, 2 or 3 [9]. Type 1 facilities offer on-site care provided by physicians, nurses and care assistants (available in Italy, the Netherlands, and Poland). Type 2 facilities offer on-site care provided by nurses and care assistants with medical provision provided by local, external primary care services (available in all countries). Type 3 facilities offer on-site care provided by care assistants, with nursing and medical provision provided by local, external primary care services (available in England). Funding status of the LTCF was classed as either public (non-profit), private (non-profit) or private (for profit).

### 2.3. Statistical Analysis 

Data were collected on 1707 deceased residents from 322 LTCFs. Residents were excluded from the sample if length of stay was less than one day or could not be calculated, if a resident was missing data on age or was younger than 65 years of age on admission, or no questionnaire was returned by LTCF staff (*n* = 470), resulting in a final sample of 1237 residents. Non-response analysis was conducted on residents for whom staff returned questionnaires and for those whom staff did not return questionnaires, based on the length of stay. Sample characteristics and frequencies for each of the outcomes are reported by length of stay.

For continuous outcomes, associations between length of stay and quality of care in the last month of life (QoD-LTC), comfort in the last week of life (EOLD-CAD) and their subscales were determined using generalised linear regression models. In each model, total scores of the QoD-LTC, EOLD-CAD and their subscales were added as the dependent variable, with length of stay added as a covariate. Resident, facility and country level characteristics previously identified as varying by length of stay were also added to each model as covariates; these were age, gender, marital status, place of admission, cancer, dementia, physical functioning, LTCF type, LTCF funding status and country. A variable identifying each LTCF was added as a random factor. Goodness of fit for each model was assessed using the Akaike information criterion. 

For binary outcomes, associations between length of stay and the presence of advance directives, contact with health services and consensus on care and treatment were determined using logistic regression models. In each model, the outcome was added as a dependent variable, with length of stay added as a covariate along with resident, facility and country level characteristics. A variable identifying each LTCF was added as a random factor. The adequacy of the model was assessed using the Hosmer and Lemeshow’s goodness-of-fit test. Interactions between age and gender were tested and added to the model where appropriate. Multi-collinearity was checked using variance inflation factors [43].

A positive coefficient indicates that an increase in the value of the dependant variable is associated with an increase in the value of the independent variable. A negative coefficient indicates that a decrease in the value of the dependant variable is associated with a decrease in the value of the independent variable. Statistical significance was set as *p* < 0.05. All analyses were performed using Stata (version 16) [44].

## 3. Results

The final sample included 1237 residents; 262 in Belgium, 252 in Finland, 192 in Italy, 193 in the Netherlands, 263 in Poland and 75 in England. No significant differences were identified in the lengths of stay of residents for whom a staff questionnaire was or was not completed and returned (*p* = 0.356). The median length of stay was 73.4 weeks (range 16–103.9 weeks) and average length of stay was 126 weeks (SD 157), ranging from 93 (SD 156) to 163 (SD 182) weeks. The mean age of residents at admission was 83.9 years (SD 7.2), ranging from 81.56 (SD 7.12) in residents with length of stay over 5 years to 85.45 (SD 7.2) in residents with a length of stay of 3 months to 1 year. The percentage of residents who were female was 67.6%, ranging from 55.8% in residents with length of stay of 1 to 3 months and 81.1% in residents with length of stay over 5 years. Characteristics of the sample and main outcomes are shown in Table 1 and Table 2. 

### 3.1. Quality of End of Life Care in the Last Month of Life (QoD-LTC)

Associations between end of life care and length of stay are shown in Table 3. Length of stay was associated with quality of care in the last month of life in the multivariate model. Total scores on the QoD-LTC were significantly higher in residents with a length of stay of 3 months to 1 year compared to under 1 month (*p* = 0.002); and increase significantly up to and over 5 years (*p* < 0.001). Scores on the personhood subscale were significantly higher in residents with a length of stay of 3 months to 1 year compared to under 1 month (*p* = 0.010); and increase significantly up to and over 5 years (*p* = 0.001). Scores on the closure subscale were also significantly higher in residents with a length of stay of 1 to 3 months compared to under 1 month (*p* = 0.014); and increase significantly up to and over 5 years (*p* < 0.001). Scores on the preparatory tasks subscale were significantly higher between 1 to 2 years (*p* = 0.027), 2 to 3 years (*p* = 0.002) and 3 to 5 years (*p* < 0.001), and approached statistical significance at over 5 years (*p* = 0.052). 

### 3.2. Comfort in the Last Week of Life (EOLD-CAD) 

Total scores on the EOLD-CAD were higher in residents with longer lengths of stay, however length of stay was significantly associated with comfort in the last week of life at only over 5 years compared to under 1 month (*p* = 0.005) in the multivariate model. Scores on the physical distress subscale were significantly higher in residents with a length of stay between 1 to 2 years (*p* = 0.040), 3 to 5 years (*p* = 0.027) and over 5 years (*p* < 0.001). Scores on the emotional distress subscale were significantly higher in residents with a length of stay of 3 to 5 years (*p* = 0.007) and over 5 years (*p* = 0.001) and on the wellbeing subscale at over 5 years (*p* = 0.001). Scores on the dying symptoms subscale were not significantly associated with length of stay.

### 3.3. Contact with Health Services in the Last Month of Life and Place of Death

Residents with a length of stay of over 5 years had significantly fewer hospital admissions in the last month of life compared to under 1 month (*p* = 0.008). No significant associations were identified between physician visits and length of stay or emergency department visits and length of stay. Death in hospital was significantly less likely compared to death in a LTCF at a length of stay of 3 to 5 years (*p* = 0.044), however no trend was identified as length of stay increased.

### 3.4. Presence of Advance Directives 

Residents were significantly more likely to have a written advance directive in place at 3 to 5 years and over 5 years, compared to under 1 month post admission (*p* = 0.002 and *p* = 0.015, respectively). Residents were also significantly more likely to have a lasting power of attorney for personal welfare in place between 1 to 2 years (*p* = 0.004), 2 to 3 years (*p* = 0.025), 3 to 5 years (*p* = 0.024) and over 5 years (*p* = 0.001), compared to under 1 month. The likelihood of a staff member having spoken with the resident about end of life preferences was significantly associated with length of stay over 5 years, compared to 1 month (*p* = 0.031). The likelihood of a staff member having spoken with a relative about end of life preferences was significantly associated with length of stay of 3 to 5 years, compared to under 1 month (*p* = 0.025).

### 3.5. Consensus on Care and Treatment in the Last Month of Life 

No significant associations were identified between length of stay and consensus on care and treatment in the last month of life among LTCF staff, among family or among all those involved in the resident’s care.

## 4. Discussion

### 4.1. Summary of Main Findings

Longer lengths of stay were associated with higher scores of quality of care in the last month of life and on the personhood, closure and preparatory tasks subscales. Longer lengths of stay were also associated with higher scores of comfort in the last week of life, on all subscales except the dying symptoms subscale. Associations between longer lengths of stay and quality of end of care occurred earlier than in comfort in the last week of life, with significantly higher scores identified from 3 months compared to 1 year. 

A slight but statistically significant association was identified with fewer hospital admissions and resident deaths in hospital when length of stay was longer. In addition, longer stay residents were more likely to have written advance directives and lasting power of attorney in place, and have had a staff member discuss end of life care with either themselves or a relative. No significant associations were identified between length of stay and physician visits, emergency department visits or consensus on care and treatment. The analysis controlled for resident characteristics associated with variation in length of stay and country of residence.

### 4.2. Strengths and Limitations

This is the first study of which the research team is aware that focuses specifically on the relationship between length of stay in an LTCF and end of life care. A strength of the data used in this analysis is their representativeness of a large sample of LTCFs across six European countries. As the study was retrospective, the data were not limited by a follow up period, therefore data on length of stay is available for residents with especially long lengths of stay (no right censoring). 

The main limitation of the study is that the data were collected by staff members up to 3 months after the resident’s death. Such an approach has a number of implications for the validity of the data. Firstly, the risk of recall bias increases, however, data collected on length of stay cannot be biased as there is no loss to follow up. In addition, if such as bias exists in this dataset as opposed to non-systematic measurement error, it would be the same across all countries, although the direction of the bias is unclear [45].

Secondly, the relationship between the staff member providing the data and the resident may affect the findings. It is possible that staff members who did not feel they knew the resident well enough to answer the questionnaire, and could not access written records on the residents care, did not return the questionnaire, leading to a bias in the data towards staff members with closer relationships with the residents. 

Further to this, one explanation for the findings could be that staff members may feel they know and understand residents with longer lengths of stay more than recently admitted residents, and are therefore more confident in their judgement of resident experience. As some of the indicators used in the EOLD-CAD are relatively subjective to judgement (fear, serenity, anxiety etc.) the findings may be influenced by greater confidence in the staff member to make these assessments, and therefore more likely to provide appropriate care, i.e., symptom management. 

There are also specific limitations to each of the measures used to indicate quality of end of life care. For example, data were not collected on the time when written advance directives or lasting power of attorney were established, therefore it is unclear if these occurred prior to LTCF admission. Discussions with the resident and relative about end of life care may have occurred, however no data were collected on whether the decisions made in these conversations were recorded or acted upon, where possible. Data collected on advance directives are specific to the availability and legality of advance directives in each country. For example, the data from England does not necessarily indicate that a conversation has occurred between LTCF staff and the resident, it is possible that advanced care planning documentation collected as part of the Gold Standard Framework was used to obtain the answer, which were neither initiated or filled in by the residents themselves [46]. Future research could further contextualise these findings by including the approach to end of life care adopted at each facility, including staff mix and training. 

Finally, the data used in this analysis is limited to consensus in care and treatment as judged only by one staff member and not family members. The analysis is limited by a lack of data collected from residents and relatives’ perspectives on their perceptions of the quality of care at end of life.

### 4.3. Interpretation of Findings 

The primary finding of this analysis is that residents who have resided in an LTCF for a longer length of time had better quality of care and comfort at end of life than recent LTCF admissions, after controlling for characteristics of short and long stay resident populations.

Differences in the findings for each of the QoD-LTC subscales require further discussion. The preparatory tasks subscale refers to activities which can be planned in advance (treatment preferences in writing, establishing a named decision-maker, funeral planning) indicating that lack of time for such activities to be enacted by LTCF staff may explain lower scores among newly admitted residents. Similarly, the personhood subscale focuses on the relationship between the resident and wider staff (a nurse or aide with whom the resident felt comfortable, affectionate touch daily, physician knew him or her as a whole person) which, again, develop over time. 

However, the items on the dying symptoms subscale of the EOLD-CAD (choking, gurgling, difficulty swallowing, shortness of breath) are arguably more difficult for LTCF staff to modify without physician involvement. Additionally, data were not collected on whether the resident received treatment for such symptoms, therefore, in this study the presence of such symptoms does not necessarily indicate poorer quality of care. 

In a review of preconditions for successful advance care planning in nursing homes, five domains were identified; sufficient knowledge and skills, willingness and ability to participate in advance care planning, a good relationship (between staff and family caregivers and residents), availability of an administrative system for documenting wishes and monitoring care and supportive contextual factors within the nursing home [47]. Applied to the findings of this paper, a longer length of residence before death could allow for the involvement of an appropriately skilled professional, for a record of resident wishes to be written and accessible or for sufficient time and resources to be allocated to establishing preferences at end of life care. However, as the association only becomes significant after 1 year of residence, a more plausible explanation could be that it takes this long for a relationship to be established between LTCF staff, residents and their families. 

The few significant results identified for consensus in care and contact with health services shows that these experiences remain consistent regardless of subsequent length of stay. Although the analysis failed to show a difference across the groups, this could indicate that if consensus is not established in the first month after admission, it is unlikely to be subsequently achieved. Alternatively, admissions to either hospital or an emergency department for preventable reasons (pneumonia, urinary tract infections etc.) are common in this population, however, the likelihood may not differ based on length of stay.

### 4.4. Implications for Future Research, Policy and Practice 

International epidemiological research on the health and health care needs of LTCF residents is gaining more attention [48,49,50], allowing for heterogeneity in the care residents’ experience to be explored further. Despite the emphasis on ageing in place [51], and a common preference for older adults to remain living in the community until death [52], there is little evidence to suggest that cohorts on admission are in poorer health or have shorter lengths of stay than those in previous years [53]. Further research is needed to explore the underlying reasons for this trend, and its implications for providing good quality end of life care to all LTCF residents. The inclusion of LTCF residents in nationally representative epidemiological studies, allowing for longitudinal analysis of characteristics prior to admission [54] and better identification of LTCF residents in existing routinely collected datasets [55], would greatly support research in this area.

Although numerous interventions to improve end of life care have been developed and implemented in LTCFs, few have tailored their approach to residents depending on length of stay. In a recent scoping review of implementation strategies for such interventions, prioritising time for staff members to provide end of life care, and ensuring staff are available for residents to develop a relationship with, allowing discussions on end of life to occur, were highlighted as facilitators to successful implementation [56]. An approach which can be tailored to shorter and longer stay residents is needed, including how such an environment can be developed prior to resident admission. In particular, further research is needed to explore the experiences of residents with lengths of stay under 1 month and the underlying mechanisms that account for fewer indicators of end of life care.

## 5. Conclusions

Older adults residing in LTCFs often have multiple health needs, are likely to be approaching end of life and require good quality end of life care. This study explored associations between length of stay in LTCF residents with five measures of end of life care, using data on deceased residents in six European countries. In addition to the differences in population characteristics of shorter and longer stay residents, the findings of this analysis indicate that residents with longer lengths of stay experience better end of life care than those with shorter lengths of stay on some of the indicators explored. This trend is identified even after controlling for resident characteristics associated with variation in length of stay and country of residence. Further research is needed to explore why such an association is found, and how appropriate end of life care can be provided to all residents from admission to death. 

## Figures and Tables

**Table 1:** Characteristics of deceased long term care facility (LTCF) residents by length of stay in a LTCF until death.

	Under 1 month	1 to 3 months	3 months to 1 year	1 year to 2 years	2 year to 3 years	3 year to 5 years	5 years +	Total	*p* value
	*n* = 163	*n* = 135	*n* = 223	*n* = 208	*n* = 162	*n* = 160	*n* = 186	*n* = 1237	
Age at admission—mean (SD)	83.8 (8.1)	83.7 (7.7)	85.5 (7.2)	84.56 (6.7)	84 (7.1)	83.67 (6)	81.6 (7.2)	83.9 (7.2)	**<0.001**
Gender—female (%)	91 (55.8)	87 (64.4)	142 (64.3)	132 (64.4)	110 (68.8)	119 (74.4)	150 (81.1)	831 (67.6)	**<0.001**
Marital status—married or in a civil partnership	47 (34.8)	37 (31.6)	47 (22.7)	47 (24.1)	35 (22.3)	27 (17.4)	13 (7.2)	253 (22.1)	**<0.001**
Place of admission (%)									
Community	54 (34.6)	49 (39.8)	87 (43.9)	82 (45.1)	68 (47.9)	80 (58.4)	112 (69.6)	532 (48.4)	**<0.001**
Hospital	78 (50)	52 (42.2)	75 (37.9)	59 (32.4)	39 (27.5)	30 (21.9)	32 (19.9)	365 (33.2)	**<0.001**
Other LTCF	24 (15.4)	22 (17.9)	36 (18.2)	41 (22.5)	35 (24.7)	27 (19.7)	17 (10.6)	202 (18.4)	**0.036**
BANS-S—total score—mean (SD) *	20.51 (5.4)	20.00 (4.9)	19.47 (4.8)	19.39 (4.7)	19.46 (4.5)	20.15 (4.3)	19.68 (4.9)	19.77 (4.8)	0.239
Cancer (%)	26 (16)	29 (21.5)	39 (17.5)	31 (14.9)	21 (13)	17 (10.6)	18 (9.7)	181 (14.6)	**0.046**
Dementia (%)									
Resident did not have dementia	63 (48.5)	39 (34.2)	56 (29)	57 (31.5)	33 (23.1)	38 (27.9)	52 (31.9)	338 (31.9)	**0.001**
Mild or moderate	11 (8.5)	24 (21.1)	31 (16.1)	25 (13.8)	17 (11.9)	11 (8.09)	15 (9.2)	134 (12.6)	**0.014**
Severe, very severe or advanced dementia	56 (43.1)	51 (44.8)	106 (54.9)	99 (54.7)	93 (65.1)	87 (64)	96 (58.9)	588 (55.5)	**0.001**
LTCF type (%)									
Type 1—onsite nursing/onsite physician	79 (48.8)	53 (39.6)	60 (27.7)	39 (19.2)	36 (22.4)	22 (13.9)	27 (14.6)	316 (25.9)	**<0.001**
Type 2—onsite nursing/offsite physician	81 (50)	79 (59)	153 (70.5)	158 (77.5)	123 (76.4)	129 (81.7)	146 (78.9)	869 (71.2)	**<0.001**
Type 3—offsite nursing/offsite physician	2 (1.2)	2 (1.5)	4 (1.8)	7 (3.4)	2 (1.2)	7 (4.4)	12 (6.5)	36 (3)	**0.023**
LTCF ownership (%)									
Public—non profit	91 (56.2)	88 (65.7)	129 (60)	120 (59.1)	95 (59.8)	101 (63.9)	121 (65.4)	745 (61.3)	0.497
Private—non profit	45 (27.8)	26 (19.4)	53 (24.7)	46 (22.7)	44 (27.7)	32 (20.3)	40 (21.6)	286 (23.5)	0.427
Private—profit	26 (16.1)	20 (14.9)	33 (15.4)	37 (18.2)	20 (12.6)	25 (15.8)	24 (13)	185 (15.2)	0.790
Country (%)									
Belgium	17 (10.4)	19 (14.1)	49 (22)	46 (22.1)	40 (24.7)	35 (21.9)	56 (30.1)	262 (21.2)	**<0.001**
Finland	25 (15.3)	27 (20)	41 (18.4)	53 (25.5)	35 (21.6)	43 (26.9)	28 (15.1)	252 (20.4)	**0.029**
Italy	29 (17.8)	19 (14.1)	39 (17.5)	37 (17.8)	26 (16.1)	25 (15.6)	17 (9.1)	192 (15.5)	0.228
Netherlands	14 (8.6)	16 (11.9)	34 (15.3)	35 (16.8)	30 (18.5)	25 (15.6)	39 (21)	193 (15.6)	**0.045**
Poland	71 (43.6)	47 (34.8)	47 (21.1)	25 (12)	22 (13.6)	20 (12.5)	31 (17)	263 (6.1)	**<0.001**
England	7 (4.3)	7 (5.2)	13 (5.8)	12 (5.8)	9 (5.6)	12 (7.5)	15 (8.1)	75 (6.1)	0.797

BANS-S: Bedford Alzheimer Nursing Severity Scale; LTCF: long term care facility; SD: standard deviation. * higher scores indicate poorer physical functioning. *p* values calculated using Pearson chi-square and one way ANOVAs. A *p* value of <0.05 indicated a statistically significant difference in values between lengths of stay. Missing data: gender *n* = 8, marital status *n* = 90, place of admission *n* = 138, BANS-S *n* = 22, dementia *n* = 177, LTCF type *n* = 16 and LTCF ownership *n* = 21.

**Table 2:** Indicators of end of life care of deceased LTCF residents by length of stay in a LTCF until death.

	Under 1 month	1 to 3 months	3 months to 1 year	1 year to 2 years	2 year to 3 years	3 year to 5 years	5 years +	Total	*p* value
	*n* = 163	*n* = 135	*n* = 223	*n* = 208	*n* = 162	*n* = 160	*n* = 186	*n* = 1237	
Quality of care in the last month of life (QoD-LTC)									
Total score—mean (SD)	35.80 (6.96)	37.75 (6.97)	38.59 (7.56)	38.89 (7.73)	39.55 (7.32)	40.06 (8.08)	40.41 (7.65)	38.79 (7.62)	**<0.001**
Personhood subscale—mean (SD)	19.67 (3.35)	20.19 (3.52)	20.54 (3.55)	20.99 (2.94)	21.17 (3.56)	21.11 (3.61)	21.09 (3.39)	20.70 (3.44)	**<0.001**
Closure subscale—mean (SD)	8.01 (2.72)	9.03 (2.77)	9.21 (2.91)	9.29 (2.84)	9.14 (2.94)	9.47 (2.96)	9.72 (9.72)	9.15 (9.15)	**<0.001**
Preparatory tasks subscale—mean (SD)	6.92 (3.41)	7.70 (3.26)	8.02 (3.80)	8.07 (3.95)	8.32 (3.76)	8.47 (4.08)	8.58 (3.74)	8.03 (3.77)	**0.001**
Comfort in the last week of life (EOLD-CAD)									
Total score—mean (SD)	29.63 (5.50)	29.46 (5.78)	30.58 (5.33)	31.07 (5.07)	30.41 (5.42)	31.20 (5.21)	31.82 (5.06)	30.67 (5.36)	**0.001**
Physical distress subscale—mean (SD)	4.29 (1.70)	4.35 (1.80)	4.77 (1.77)	4.85 (1.66)	4.75 (1.64)	4.76 (1.81)	5.16 (1.57)	4.73 (1.72)	**<0.001**
Dying symptoms subscale—mean (SD)	6.86 (2.47)	6.97 (2.24)	7.07 (2.12)	7.15 (1.99)	7.04 (2.04)	7.05 (2.13)	7.17 (2.22)	7.05 (2.17)	0.874
Emotional distress subscale—mean (SD)	8.90 (2.69)	9.01 (2.47)	9.36 (2.27)	9.54 (2.17)	9.55 (2.08)	9.69 (2.13)	9.88 (1.85)	9.44 (2.25)	**0.001**
Wellbeing subscale—mean (SD)	5.02 (1.90)	5.23 (1.71)	5.64 (1.81)	5.83 (1.91)	5.68 (1.86)	5.82 (1.91)	6.09 (1.78)	5.65 (1.87)	**0.001**
Contact with health services in the last month of life									
Physician visits (%)									
0–5 visits	56 (51.38)	53 (54.64)	94 (58.75)	101 (70.63)	65 (61.90)	68 (55.74)	78 (60.47)	515 (59.54)	0.055
More than five visits	53 (48.62)	44 (45.36)	66 (41.25)	42 (29.37)	40 (38.10)	54 (44.26)	51 (39.53)	350 (40.46)	
Hospital admissions (%)									
None	98 (70.50)	98 (79.03)	164 (77.36)	159 (80.71)	125 (81.70)	126 (82.35)	157 (86.26)	927 (79.91)	**0.028**
One or more visits	41 (29.50)	26 (20.97)	48 (22.64)	38 (19.29)	28 (18.30)	27 (17.65)	25 (13.74)	233 (87.14)	
Emergency department visits (%)									
None	124 (88.57)	107 (86.29)	185 (86.85)	160 (82.47)	138 (90.20)	135 (87.10)	161 (89.44)	1010 (87.14)	0.396
One or more visits	16 (11.43)	17 (13.71)	28 (13.15)	34 (17.53)	15 (9.80)	20 (12.90)	19 (10.56)	149 (12.86)	
Place of death (%)									
LTCF	133 (84.7)	106 (82.8)	195 (88.6)	173 (84.8)	139 (86.9)	145 (90.6)	159 (84)	1050 (86.5)	0.460
Hospital	24 (15.3)	22 (17.2)	25 (11.4)	31 (15.2)	21 (13.1)	15 (9.4)	26 (14.1)	164 (13.5)	
Presence of advance directives									
Resident had any written advance directives in place (%)	33 (20.3)	46 (34.07)	81 (36.3)	81 (38.9)	60 (37)	74 (46.3)	88 (47.3)	463 (37.4)	**<0.001**
Resident had lasting power of attorney for personal welfare (%)	38 (28.2)	39 (36.5)	57 (32.4)	63 (37.8)	44 (35.8)	42 (32.1)	62 (43.4)	345 (35.1)	0.182
Staff spoke with the resident about end of life care (%)	20 (13.5)	28 (22.3)	50 (23.4)	51 (24.7)	39 (24.7)	39 (25.2)	60 (33.6)	287 (24.7)	**0.005**
Staff spoke with the relative about end of life care (%)	70 (44.9)	55 (43)	129 (60.6)	128 (61.9)	105 (66.9)	112 (71.4)	110 (60.8)	709 (59.2)	**<0.001**
Consensus in care and treatment									
Among staff (%)	152 (97.4)	125 (99.2)	203 (98.1)	193 (97.5)	152 (99.4)	146 (97.3)	172 (98.9)	1143 (98.2)	0.673
Among family (%) *	142 (98.6)	108 (97.3)	187 (96.9)	183 (97.7)	147 (99.3)	137 (97.2)	158 (100)	1062 (98.2)	0.299
Among all involved (%)	152 (98.7)	120 (97.6)	200 (98.1)	194 (97.5)	152 (99.4)	146 (98)	173 (98.9)	1137 (98.3)	0.836

QoD-LTC: Quality of Dying in Long-Term Care. EOLD-CAD: End of Life in Dementia Scale-Comfort Assessment while Dying. Theoretical range of QoD-LTC—total score: 11–55, personhood subscale: 5–25, closure subscale: 3–15, preparatory tasks: 3–15. Theoretical range of EOLD-CAD—total score: 14–42, physical distress subscale: 4–12, dying symptoms subscale: 4–12, dying symptoms subscale: 4–12, emotional distress: 4–12, wellbeing subscale: 3–9. Theoretical ranges based on no missing data. LTCF: long term care facility; SD: standard deviation. *p* values calculated using Pearson chi-square and one way ANOVAs. A *p* value of <0.05 indicated a statistically significant difference in values between lengths of stay. Missing data: QoD-LTC total score *n* = 41, personhood subscale *n* = 13, closure subscale *n* = 18, preparatory tasks subscale *n* = 32, EOLD-CAD total score *n* = 76, physical distress *n* = 43, dying symptoms *n* = 42, emotional distress *n* = 50, wellbeing subscale *n* = 61, physician visits *n* = 372, hospital admissions *n* = 77, emergency department visits *n* = 78, place of death *n* = 23, lasting power of attorney *n* = 255, staff member spoke with the resident about end of life care *n* = 49, staff member spoke with the relative about end of life care *n* = 38, consensus of care among staff *n* = 73, consensus of care among family *n* = 89, consensus of care among all involved *n* = 80. * family were not involved in residents care *n* = 66.

**Table 3:** Associations between indicators of end of life care of deceased LTCF and length of stay in a LTCF until death.

	Length of Stay—Coefficient (95% CI)						
	Under 1 month	1 to 3 months	3 months to 1 year	1 year to 2 years	2 year to 3 years	3 year to 5 years	5 years +
Quality of care in the last month of life (QoD-LTC)							
Total score	ref	1.14 (–0.77–3.05)	2.71 (1.00–4.42) **	3.03 (1.23–4.84) **	4.03 (2.15–5.90) **	4.80 (2.87–6.72) **	4.16 (2.21–6.11) **
Personhood subscale	ref	0.48 (–0.39–1.36)	1.02 (0.24–1.80) *	1.34 (0.53–2.15)**	1.31 (0.47–2.15) **	1.53 (0.68–2.38) **	1.41 (0.56–2.26) **
Closure subscale	ref	0.91 (0.18–1.64)	1.21 (0.56–1.86) *	1.32 (0.65–1.99) **	1.26 (0.57–1.96) **	1.64 (0.94–2.34) **	1.72 (1.02–2.41) **
Preparatory tasks subscale	ref	0.20 (–0.73–1.14)	0.72 (–0.12–1.56)	1.00 (0.11–1.88) *	1.45 (0.53–2.37) **	1.69 (0.75–2.63) **	0.94 (–0.01–1.90)
Comfort in the last week of life (EOLD-CAD)							
Total score	ref	–0.36 (–1.75–1.04)	0.15 (–1.11–1.40)	0.66 (–0.62–1.93)	0.31 (–1.02–1.64)	1.14 (–0.21–2.49)	1.88 (0.58–3.18) **
Physical distress subscale	ref	0.16 (–0.25–0.58)	0.35 (–0.03–0.73)	0.41 (0.02–0.79) *	0.34 (–0.06–0.75)	0.46 (0.05–0.87) *	0.86 (0.46–1.25) **
Dying symptoms subscale	ref	0.05 (–0.48–0.59)	–0.10 (–0.58–0.38)	–0.15 (–0.63–0.33)	–0.07 (–0.58–0.44)	–0.01 (–0.53–0.50)	0.06 (–0.44–0.55)
Emotional distress subscale	ref	0.10 (–0.46–0.65)	0.31 (–0.19–0.81)	0.34 (–0.16–0.85)	0.49 (–0.05–1.02)	0.74 (0.20–1.28) **	0.88 (0.36–1.39) **
Wellbeing subscale	ref	0.03 (–0.42–0.48)	0.26 (–0.14–0.66)	0.40 (–0.01–0.82)	0.29 (–0.14–0.72)	0.41 (–0.02–0.85)	0.73 (0.31–1.15) **
Contact with health services in the last month of life							
Physician visits (0–5 visits vs. more than five visits)	ref	0.15 (–0.49–0.80)	0.02 (–0.57–0.62)	–0.47 (–1.12–0.18)	–0.09 (–0.76–0.58)	0.13 (–0.50–0.77)	–0.29 (–0.92–0.35)
Hospital visits (none vs. one or more visits)	ref	–0.58 (–1.33–0.16)	–0.31 (–0.96–0.34)	–0.53 (–1.22–0.17)	–0.51 (–1.23–0.21)	–0.67 (–1.40–0.07)	–1.01 (–1.76–0.26) **
Emergency department admissions (none vs. one or more visits)	ref	0.34 (–0.50–1.17)	0.11 (–0.66–0.88)	0.30 (–0.46–1.06)	–0.04 (–0.88–0.81)	0.07 (–0.76–0.90)	–0.12 (–0.94–0.71)
Place of death (LTCF vs. hospital)	ref	0.30 (–0.57–1.17)	–0.50 (–1.33–0.33)	–0.29 (–1.13–0.55)	–0.15 (–1.03–0.73)	–1.03 (–2.04–0.03)*	–0.72 (–1.66–0.22)
Presence of advance directives							
Resident had any written advance directives in place	ref	0.58 (–0.17–1.33)	0.60 (–0.09–1.29)	0.67 (–0.02–1.37)	0.58 (–0.14–1.30)	1.21 (0.46–1.96) **	0.91 (0.18–1.64) *
Resident had lasting power of attorney for personal welfare	ref	0.60 (–0.01–1.21)	0.53 (–0.04–1.10)	0.87 (0.28–1.46) **	0.72 (0.09–1.36) *	0.73 (0.10–1.36) *	1.10 (0.48–1.73) **
Staff spoke with the resident about end of life care	ref	0.66 (–0.14–1.45)	0.49 (–0.25–1.23)	0.64 (–0.11–1.38)	0.54 (–0.23–1.31)	0.65 (–0.15–1.45)	0.86 (0.08–1.64) *
Staff spoke with the relative about end of life care	ref	–0.46 (–1.07–0.16)	0.25 (–0.30–0.81)	0.14 (–0.43–0.71)	0.51 (–0.10–1.11)	0.72 (0.09–1.35) *	0.19 (–0.44–0.81)
Consensus in care and treatment							
Among staff (no vs. yes)	ref	1.00 (–1.37–3.37)	0.82 (–0.83–2.47)	0.42 (–1.18–2.02)	2.16 (–0.24–4.55)	0.87 (–1.06–2.80)	1.06 (–0.90–3.01)
Among family (no vs. yes)	ref	–0.63 (–2.48–1.23)	–0.57 (–2.30–1.17)	–0.08 (–1.99–1.83)	0.75 (–1.75–3.26)	–0.52 (–2.47–1.44)	n/a
Among all involved (no vs. yes)	ref	–0.85 (–3.34–1.65)	–0.92 (–3.25–1.40)	–0.86 (–3.20–1.48)	0.30 (–2.62–3.21)	0.07 (–2.84–2.98)	–0.50 (–3.08–2.08)

QoD-LTC: Quality of Dying in Long-Term Care. EOLD-CAD: End of Life in Dementia Scale-Comfort Assessment while Dying. LTCF: long term care facility. CI: confidence interval. Generalised mixed models with each end of life care outcome as the dependant variables, length of stay as the independent variable, age, gender, marital status, place of admission, cancer, dementia, physical functioning, LTCF type, LTCF funding status and country as covariates and a variable identifying each LTCF was added as a random factor. *p* value < 0.05 *, *p* value < 0.01 **.
